# Genetic variants of cell cycle pathway genes predict disease‐free survival of hepatocellular carcinoma

**DOI:** 10.1002/cam4.1067

**Published:** 2017-06-22

**Authors:** Shun Liu, Tian‐Bo Yang, Yue‐Li Nan, An‐Hua Li, Dong‐Xiang Pan, Yang Xu, Shu Li, Ting Li, Xiao‐Yun Zeng, Xiao‐Qiang Qiu

**Affiliations:** ^1^ Department of Epidemiology School of Public Health Guangxi Medical University 22 Shuangyong Road Nanning Guangxi 530021 China; ^2^ Affiliated Tumor Hospital of Guangxi Medical University 71 Hedi Road Nanning Guangxi 530021 China; ^3^ Shenzhen Longhua Center for Chronic Diseases Prevention and Control 118 Guanlan Road Shenzhen Guangdong 518110 China; ^4^ GuangXi Center for Disease Prevention and Control 18 Jinzhou Road Nanning Guangxi 530021 China; ^5^ Medical Scientific Research Centre Guangxi Medical University 22 Shuangyong Road Nanning Guangxi 530021 China

**Keywords:** Cell cycle pathway, hepatocellular carcinoma, single‐nucleotide polymorphism, survival

## Abstract

Disruption of the cell cycle pathway has previously been related to development of human cancers. However, associations between genetic variants of cell cycle pathway genes and prognosis of hepatocellular carcinoma (HCC) remain largely unknown. In this study, we evaluated the associations between 24 potential functional single nucleotide polymorphisms (SNPs) of 16 main cell cycle pathway genes and disease‐free survival (DFS) of 271 HCC patients who had undergone radical surgery resection. We identified two SNPs, i.e., *SMAD3* rs11556090 A>G and *RBL2* rs3929G>C, that were independently predictive of DFS in an additive genetic model with false‐positive report probability (FPRP) <0.2. The *SMAD3* rs11556090G allele was associated with a poorer DFS, compared with the A allele [hazard ratio (HR) = 1.46, 95% confidential interval (95% CI) = 1.13–1.89, *P *=* *0.004]; while the *RBL2* rs3929 C allele was associated with a superior DFS, compared with the G allele (HR = 0.74, 95% CI = 0.57–0.96, *P* = 0.023). Additionally, patients with an increasing number of unfavorable genotypes (NUGs) of these loci had a significant shorter DFS (*P*_trend_ = 0.0001). Further analysis using receiver operating characteristic (ROC) curves showed that the model including the NUGs and known prognostic clinical variables demonstrated a significant improvement in predicting the 1‐year DFS (*P *=* *0.011). Moreover, the *RBL2* rs3929 C allele was significantly associated with increased mRNA expression levels of *RBL2* in liver tissue (*P *=* *1.8 × 10^−7^) and the whole blood (*P *=* *3.9 × 10^−14^). Our data demonstrated an independent or a joint effect of *SMAD3* rs11556090 and *RBL2* rs3929 in the cell cycle pathway on DFS of HCC, which need to be validated by large cohort and biological studies.

## Introduction

Hepatocellular carcinoma (HCC) is one of the most frequently diagnosed cancers and the leading causes of cancer death worldwide. As a high incidence area of HCC, China accounts for about 50% of the total number of cases and deaths worldwide 1, 2. In 2015, it had an estimated of 466,100 and 422,100 patients is diagnosed and die of HCC in China, respectively^1^, which makes HCC a major public health concern in China. For the past decade, cancer staging systems, such as the Barcelona Clinic Liver Cancer (BCLC) staging system and Cancer of the Liver Italian Program (CLIP) score, have been established for the prognosis assessment by using clinical variables mainly including tumor size, number of primary tumors, portal vein tumor thrombosis, and inflammatory degree 3, 4. But none of these systems has been universally adopted by clinicians. Many novel molecular biomarkers, such as Alpha‐fetoprotein, circulating tumor cells and circulating microRNAs, have been also applied to predict HCC progression 5. However, limitations of low sensitivity and specificity are still associated with these biomarkers when used in disease staging 5. Long‐term outcome prediction for HCC patients remains unfavorable 6, 7, which hampers the development of personalized clinical assessment for HCC patients and calls for identifying additional, more discriminant prognostic indicators.

In recent years, the development of human genome research has been witnessed much success in mining cancer‐related germline genetic variants, especially single nucleotide polymorphisms (SNPs). The current advance in genome‐wide association studies (GWASs) has led to the identification of several SNPs that are associated with HCC risk 8, 9, 10, 11. Additionally, a series of studies also reported that the HCC patients survival was associated with SNPs in genes functioning in pivotal biological pathways or processes, such as cytokine genes 12, 13, telomere maintenance genes 14, DNA repair genes 15, Wnt/β‐catenin pathway genes 16. These pieces of evidence demonstrate that genetic variants act as indicators of different processes of HCC and may provide further information beyond current clinical staging and pathologic prognostic assessments.

The cell cycle is a set of organized and monitored events responsible for proper cell division into two daughter cells, which includes four traditional subdivision phases (i.e., G1, S, G2 and M) and is controlled by the signaling pathway comprising genes encoding cyclins, cyclin‐dependent kinases, and cyclin kinase inhibitors as well as the related regulators (e.g., MYC) 17, 18, 19. Disruption of the cell cycle pathway can result in cell cycle arrest and has previously been related to prognosis of human cancers 20, 21. For example, CDKN2A and RB1 expression levels have been associated with survival of patients with advanced‐stage ovarian cancer 22. In addition, several studies have also demonstrated that aberrant expression of cell cycle pathway genes, including cyclin (A, D1 and E), CDC2, p27 and p21, is associated with prognosis for patients with HCC 23, 24, 25, 26, 27.

We have previously investigated the association of genetic variants in cell cycle pathway genes with susceptibility to HCC and found that SNPs in *MCM4*,* CHEK1* and *KAT2B* were associated with HCC risk 28. There are additional studies demonstrating the associations between genetic variants of cell cycle pathway genes and survival of cancers of the ovaries, oral cavity and lung 29, 30, 31. However, the associations between genetic variants of cell cycle pathway genes and prognosis of HCC remain largely unknown. In this study, we hypothesized that genetic variants in cell cycle pathway genes are associated with survival of HCC patients. To test this hypothesis, we evaluated the associations of potential functional SNPs in 16 main cell cycle pathway genes (*CDC25C*,* CDC7*,* CDKN1A*,* CDKN2A*,* CHEK1*,* MCM4*,* MCM7*,* MYC*,* ORC6L*,* KAT2B*,* PLK1*,* RAD21*,* RBL2*,* SMAD3*,* TGFB3*, and *YWHAB*) with clinical outcomes of 271 HCC cases who had undergone radical surgery resection.

## Materials and Methods

### Study population

The patients were derived from our previously published case–control study 28. A total of 1127 patients with newly diagnosed and pathologically confirmed HCC were recruited consecutively between June 2007 and December 2013 from the First Affiliated Hospital and Affiliated Tumor Hospital of Guangxi Medical University. Among these patients, 271 undergone radical surgery resection with completed clinical information and long‐term follow‐up data for survival analysis. Clinical information (including age at diagnosis, sex, ethnicity, hepatitis B virus infection, smoking status, drinking status, capsule, portal vein tumor thrombosis, cirrhosis, and BCLC staging) was collected from medical records. Peripheral venous blood was collected in a vacuum EDTA anticoagulant tube from each participant. Genomic DNA was extracted using a standard phenol/chloroform extraction method and stored at −80°C. The study protocol was approved by the ethical committee of Guangxi Medical University, and a written informed consent was obtained from each of the participants.

### Follow‐up

All of the patients were followed up to collect data on postoperative recurrence, distant metastasis and death condition through phone calls or the medical records. The endpoint was disease‐free survival (DFS). DFS was defined as the interval between the date of tumor resection and the date of the presence of relapse/metastasis/death or until the last follow‐up. Patients who were lost to follow‐up, who were alive at the last follow‐up and who did not have developed recurrence/metastasis were censored.

### SNP selection and genotyping

The methods of SNP selection and genotyping have been described previously 28. In brief, we found 3826 differential expressed genes by analysis of three HCC‐related genome expression microarrays (GSE14520, GSE25097, and GSE12941) from GEO database. After performing the gene ontology classification and pathway enrichment analysis by using blast2GO and DAVID (https://david.ncifcrf.gov/), we identified 40 cell cycle pathway genes involved in the cellular process. Considering the literature on these genes with cancer survival, we selected 16 genes (*CDC25C*,* CDC7*,* CDKN1A*,* CDKN2A*,* CHEK1*,* MCM4*,* MCM7*,* MYC*,* ORC6L*,* KAT2B*,* PLK1*,* RAD21*,* RBL2*,* SMAD3*,* TGFB3*, and *YWHAB*) for further analysis. The genotype information of the 16 genes were downloaded from Hapmap website (http://hapmap.ncbi.nlm.nih.gov/), and functional SNPs were selected using SNPinfo (https://snpinfo.niehs.nih.gov/) 32. Considering the minor allele frequency (MAF ≥ 0.05), linkage disequilibrium (LD, *r* ^2^ > 0.8) and potential functions in transcription factor‐binding, microRNA‐binding and splicing sites, we finally included 24 SNPs with putative functions in this study (Table 1). Then, we performed the genotyping by using a MassARRAY system (Sequenom, San Diego, CA, USA) and a matrix‐assisted laser desorption ionization‐time of flight mass spectrometry method according to the manufacturer's instructions. All the primers for PCR were designed using the Assay Designer software package of the Sequenom system (San Diego, CA, USA).

**Table 1:** SNPs selection and prediction function in SNPinfo

Genes	SNP	CHR	Position	Allele^a^	MAF	Prediction function in SNPinfo^b^
*CDC25C*	rs3734166	5	137693222	A/G	0.42	Nonsynonymous variant
*CDC7*	rs13447539	1	91758416	A/G	0.05	Splicing site and nonsynonymous variant
*CDKN1A*	rs3176320	6	36754766	A/G	0.24	Transcription factor binding site
*CDKN1A*	rs3176329	6	36755441	G/T	0.09	Transcription factor binding site
*CDKN2A*	rs3088440	9	21958159	G/A	0.12	Transcription factor and microRNA‐binding site
*CHEK1*	rs3731399	11	125002457	T/C	0.17	Transcription factor binding site
*CHEK1*	rs515255	11	125002355	C/T	0.40	Transcription factor binding site
*MCM4*	rs2305952	8	49037162	T/C	0.17	Transcription factor binding site
*MCM7*	rs2070215	7	99534733	A/G	0.17	Splicing site and nonsynonymous variant
*MCM7*	rs2261360	7	99530929	C/A	0.40	Transcription factor and microRNA‐binding site
*MCM7*	rs4928	7	99528626	C/G	0.06	Splicing site
*MYC*	rs4645948	8	128817680	C/T	0.13	Transcription factor binding site
*ORC6L*	rs12596237	16	45284886	C/T	0.05	Transcription factor binding site
*ORC6L*	rs33994299	16	45281099	T/C	0.06	Transcription factor binding site and splicing site
*PCAF*	rs17006625	3	20136100	A/G	0.14	Splicing site and nonsynonymous variant
*PCAF*	rs4858770	3	20169427	C/T	0.47	MicroRNA‐binding site
*PLK1*	rs2230915	16	23608959	G/A	0.05	Splicing site and microRNA‐binding variant
*RAD21*	rs16889105	8	117952045	A/G	0.40	Transcription factor and microRNA‐binding site
*RAD21*	rs6987652	8	117951462	G/A	0.16	Transcription factor binding site
*RBL2*	rs3929	16	52081809	G/C	0.21	MicroRNA‐binding site
*SMAD3*	rs11556090	15	65273437	A/G	0.11	MicroRNA‐binding site
*SMAD3*	rs8025774	15	65270330	C/T	0.50	MicroRNA‐binding site
*TGFB3*	rs3917148	14	75516274	A/C	0.14	Transcription factor binding site
*YWHAB*	rs2425675	20	42968348	G/A	0.18	Transcription factor binding site

SNP, single‐nucleotide polymorphism; CHR, chromosome; MAF, minor allele frequency.

a Reference allele/minor allele.

b SNPinfo: https://snpinfo.niehs.nih.gov/.

### Statistical methods

Cox proportional hazards regression analysis was conducted to assess the associations between SNPs and DRS in an additive model with adjustment for age at diagnosis, sex, ethnic, hepatitis B virus infection, smoking status, drinking status, capsule, portal vein tumor thrombosis, cirrhosis and BCLC staging. The false‐positive report probability (FPRP) approach was applied to correct for multiple comparisons 33. We assigned a prior probability of 0.1 to detect an HR of 2.0 for the adverse genotypes and alleles of SNPs with an elevated risk. Only the significant results with FPRP <0.2 were considered noteworthy. The stepwise Cox model including noteworthy SNPs and clinical variables was conducted to choose independent predictive SNPs. Kaplan–Meier estimation of survival functions and Log‐rank tests were used to evaluate the single and combined effects of risk genotypes on DFS. The ROC curve was used to evaluate the performance of different prediction models, and Delong's test was conducted to compare the area under the curves (AUC) across different models 34. Finally, to assess the correlation between genetic variants and mRNA expression of the corresponding genes, the expression quantitative trait loci (eQTL) analysis was also performed using data from the GTEx project (http://www.gtexportal.org/home/) and the HapMap3 Project (Release version 2) by general linear regression model in additive genetic model 35, 36. All analyses were performed using SAS (version 9.1.3; SAS Institute, Cary, NC), unless otherwise specified.

## Results

### Basic characteristics of study populations

The details of clinical information about age at diagnosis, sex, ethnicity, hepatitis B virus infection, smoking status, drinking status, capsule, portal vein tumor thrombosis, cirrhosis status, BCLC staging and survival outcomes are showed in Table 2. There were 271 cases of staging 0‐C (BCLC staging) who had undergone radical surgery resection with a median follow‐up time of 19.0 months. At the time of analysis, 185 (68.3%) patients reached the DFS endpoint. The median DFS time was 20.3 months and 1, 3 and 5‐year survival rate were 55.4%, 40.2%, and 28.9%, respectively. Univariate analysis indicated that only portal vein tumor thrombosis, cirrhosis, and BCLC staging were significantly associated with HCC DFS (*P *=* *0.007, 0.030 and 0.004, respectively).

**Table 2:** Association between characteristic factors and HCC DFS by univariate Cox proportional hazards regression model

Factors	Patients	Event (%)^a^	HR (95% CI)	*P*
Sex				
Female	24	19 (79.2)	1.00	
Male	247	166 (67.2)	0.99 (0.62–1.59)	0.964
Age				
≤50	164	112 (68.3)	1.00	
>50	107	73 (68.2)	0.88 (0.66–1.18)	0.403
Ethnicity				
Han	175	125 (71.4)	1.00	
Zhuang/others	96	60 (62.5)	0.85 (0.62–1.15)	0.285
HBsAg				
Negative	32	22 (68.8)	1.00	
Positive	239	163 (68.2)	1.00 (0.64–1.56)	0.987
Smoking				
Never	175	120 (68.6)	1.00	
Ever	96	65 (67.7)	1.11 (0.82–1.50)	0.503
Drinking				
Never	173	118 (68.2)	1.00	
Ever	98	67 (68.4)	1.09 (0.81–1.47)	0.587
Intact capsule				
No	226	152 (67.3)	1.00	
Yes	45	33 (73.3)	1.11 (0.77–1.62)	0.573
Portal vein tumor thrombosis				
No	214	139 (65)	1.00	
Yes	57	46 (80.7)	1.59 (1.13–2.22)	0.007
Cirrhosis				
No	98	75 (76.5)	1.00	
Yes	173	110 (63.6)	0.72 (0.54–0.97)	0.030
BCLC staging				
0/A	86	50 (58.1)	1.00	
B/C	176	128 (72.7)	1.62 (1.17–2.25)	0.004
Missing	9			

HCC, hepatocellular carcinoma; DFS, disease‐free survival; HRs, hazards ratio; CI, confidence interval; HBsAg, hepatitis B virus surface antigens; BCLC, Barcelona Clinic Liver Cancer.

a Including relapse, metastasis and death.

### Survival analysis of SNPs and DFS

**Figure 1:**
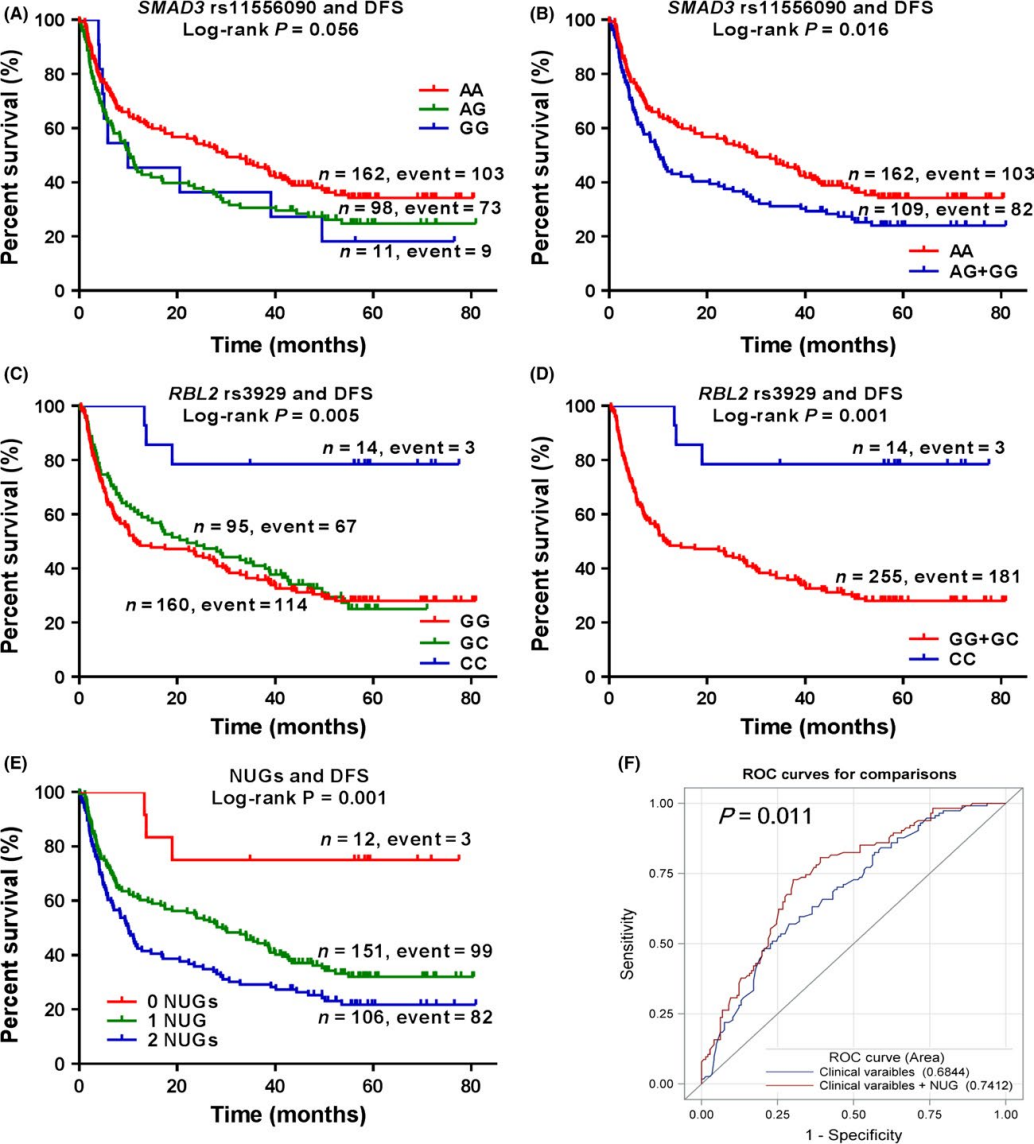
*SMAD3* rs11556090 and *RBL2* rs3929 associated with disease‐free survival of hepatocellular carcinoma patients. (A–D) Kaplan–Meier survival curves of single nucleotide polymorphisms in different genetic models: rs11556090 in an additive genetic model (A) and dominant model (B); rs3929 in an additive genetic model (C) and recessive model (D). (E) Kaplan–Meier survival curves of combined effects of the unfavorable genotypes. (F) Receiver operating characteristic (ROC) curve and area under the curve (AUC) estimation for prediction of 1‐year DFS.

As shown in Table 3, 16 SNPs were genotyped successfully in all HCC patients, while the other eight SNPs had a few missing data. Because *ORC6L* rs12596237 had only one single genotype, we included 23 SNPs for further analysis. We first performed Cox proportional hazards regression analysis to assess associations of individual SNPs in genes of the cell cycle pathway with DFS. We found that four SNPs were significantly associated with DFS at *P *<* *0.05 under an additive genetic model, of which three SNPs, i.e. rs11556090 (*P *=* *0.004, FPRP = 0.03), rs8025774 (*P *=* *0.020, FPRP = 0.15) and rs3929 (*P *=* *0.023, FPRP = 0.17), had FPRP <0.2. To assess the independent prognostic factor of DFS, we further conducted stepwise Cox regression analysis by including the clinical variables and the significant SNPs. The results demonstrated that BCLC staging, *SMAD3* rs11556090, and *RBL2* rs3929 were still significantly associated with DFS of HCC patients (Table 4). Taken all together, we selected *SMAD3* rs11556090 A>G and *RBL2* rs3929G>C as the final independent SNPs for further analyses. The identified SNPs were both predicted to be in the miRNA‐binding sites (Table 1), which may affect the binding capacity of miRNA and the corresponding gene. As shown in Table 5, our results demonstrated that the *SMAD3* rs11556090G allele was significantly associated with a shorter DFS (trend test: *P *=* *0.004), while the *RBL2* rs3929 C allele was associated with a superior DFS (trend test: *P *=* *0.023). Similarly, the *SMAD3* rs11556090 GG+GC genotypes were associated with a shorter DFS, compared with the CC genotype (HR = 1.54, 95% CI = 1.13–2.09, *P *=* *0.006), and similar results were found for the *RBL2* rs3929 CC+GC genotypes, compared with the GG genotype (HR = 4.98, 95% CI = 1.57–15.83, *P *=* *0.006). For the visual effect, we used Kaplan–Meier curves to depict associations between the significant SNPs and DFS (Fig. 1A–D).

**Table 3:** Association between SNPs of cell cycle pathway genes and HCC DFS by multivariate Cox proportional hazards regression model

SNP	Gene	Allele^a^	Frequency of genotypes (%)			Missing	DFS		
			MhoG	HetG	RhoG		HR (95% CI)^b^	*P* b	FPRP
rs3734166	*CDC25C*	A/G	108 (39.9)	136 (50.2)	27 (10)	–	0.92 (0.73–1.16)	0.460	0.81
rs13447539	*CDC7*	A/G	260 (95.9)	11 (4.1)	–	–	0.69 (0.28–1.73)	0.433	0.84
rs3176320	*CDKN1A*	A/G	145 (53.5)	112 (41.3)	14 (5.2)	–	0.91 (0.70–1.17)	0.445	0.80
rs3176329	*CDKN1A*	G/T	177 (65.3)	73 (26.9)	7 (2.6)	4 (1.5)	1.12 (0.83–1.50)	0.466	0.81
rs3088440	*CDKN2A*	G/A	193 (71.2)	73 (26.9)	5 (1.8)	–	0.86 (0.63–1.19)	0.370	0.77
rs3731399	*CHEK1*	T/C	206 (76.0)	59 (21.8)	5 (1.8)	1 (0.4)	1.22 (0.89–1.68)	0.224	0.67
rs515255	*CHEK1*	C/T	106 (39.1)	129 (47.6)	31 (11.4)	5 (1.8)	1.01 (0.81–1.26)	0.938	0.89
rs2305952	*MCM4*	T/C	195 (72.0)	74 (27.3)	2 (0.7)	–	1.02 (0.74–1.41)	0.917	0.89
rs2070215	*MCM7*	A/G	123 (45.4)	110 (40.6)	38 (14)	–	1.08 (0.88–1.33)	0.464	0.81
rs2261360	*MCM7*	C/A	117 (43.2)	111 (41.0)	42 (15.5)	1 (0.4)	0.99 (0.80–1.22)	0.910	0.89
rs4928	*MCM7*	C/G	243 (89.7)	27 (10.0)	1 (0.4)	–	0.93 (0.57–1.54)	0.782	0.88
rs4645948	*MYC*	C/T	192 (70.8)	74 (27.3)	5 (1.8)	–	0.95 (0.70–1.30)	0.744	0.87
rs12596237	*ORC6L*	C/T	271 (100)	–	–	–	–	–	–
rs33994299	*ORC6L*	T/C	254 (93.7)	16 (5.9)	–	1 (0.4)	0.44 (0.21–0.96)	0.039	0.48
rs17006625	*PCAF*	A/G	153 (56.5)	100 (36.9)	18 (6.6)	–	1.03 (0.81–1.30)	0.823	0.88
rs4858770	*PCAF*	C/T	99 (36.5)	130 (48.0)	38 (14.0)	4 (1.5)	0.98 (0.77–1.24)	0.861	0.89
rs2230915	*PLK1*	G/A	251 (92.6)	19 (7.0)	1 (0.4)	–	1.09 (0.65–1.83)	0.742	0.87
rs16889105	*RAD21*	A/G	114 (42.1)	112 (41.3)	45 (16.6)	–	1.17 (0.94–1.45)	0.157	0.59
rs6987652	*RAD21*	G/A	199 (73.4)	66 (24.4)	6 (2.2)	–	0.88 (0.65–1.21)	0.438	0.80
rs3929	*RBL2*	G/C	160 (59.0)	95 (35.1)	14 (5.2)	2 (0.7)	0.74 (0.57–0.96)	0.023	0.17
rs11556090	*SMAD3*	A/G	162 (59.8)	98 (36.2)	11 (4.1)	–	1.46 (1.13–1.89)	0.004	0.03
rs8025774	*SMAD3*	C/T	79 (29.2)	141 (52.0)	50 (18.5)	1 (0.4)	0.76 (0.61–0.96)	0.020	0.15
rs3917148	*TGFB3*	A/C	212 (78.2)	56 (20.7)	2 (0.7)	1 (0.4)	0.97 (0.69–1.38)	0.872	0.89
rs2425675	*YWHAB*	G/A	173 (63.8)	88 (32.5)	10 (3.7)	–	1.01 (0.77–1.33)	0.919	0.89

SNP, single‐nucleotide polymorphism; HCC, hepatocellular carcinoma; DFS, disease‐free survival; MhoG, major homozygous genotype; HetG, heterozygous genotype; RhoG, rare homozygous genotype; HR, hazards ratio; CI, confidence interval; FPRP, false positive report probability.

a Reference allele/minor allele.

b Adjusted by age, sex, ethnicity, HBsAg status, smoking status, drinking status, intact capsule, portal vein tumor thrombosis, cirrhosis and BCLC staging.

**Table 4:** Predictors of DFS obtained from stepwise Cox proportional hazards regression analysis

Parameter	Parameter estimate	Standard error	Chi‐square	HR (95% CI)^a^	*P* a
Age	−0.11	0.16	0.45	0.90 (0.66–1.22)	0.501
Sex	0.02	0.25	0.01	1.02 (0.63–1.67)	0.930
Ethnicity	−0.13	0.15	0.77	0.88 (0.65–1.18)	0.380
BCLC staging	0.45	0.14	9.73	1.57 (1.18–2.08)	0.002
rs3929	−0.27	0.13	4.39	0.76 (0.59–0.98)	0.036
rs11556090	0.30	0.13	5.49	1.36 (1.05–1.75)	0.019

DFS, disease‐free survival; HR, hazards ratio; CI, confidence interval; BCLC, Barcelona Clinic Liver Cancer.

a Stepwise analysis included sex, age, ethnicity, HBsAg status, smoking status, drinking status, intact capsule, portal vein tumor thrombosis, cirrhosis, BCLC staging, *RBL2* rs3929, *SMAD3* rs8025774, and *SMAD3* rs11556090.

**Table 5:** Association of *SMAD3* rs11556090 and *RBL2* rs3929 with DFS of HCC patients

Genotypes	Patients	Events (%)^a^	Univariate analysis		Multivariate analysis^b^	
			HR (95%CI)	*P*	HR (95%CI)	*P*
*SMAD3* rs11556090						
AA	162	103 (63.6)	1		1	
AG	98	73 (74.5)	1.42 (1.05–1.92)	0.021	1.50 (1.09–2.06)	0.013
GG	11	9 (81.8)	1.45 (0.73–2.86)	0.289	2.00 (0.98–4.05)	0.056
Trend test				0.017		0.004
AA	162	103 (63.6)	1		1	
AG+GG	109	82 (75.2)	1.32 (1.04–1.67)	0.023	1.54 (1.13–2.09)	0.006
*RBL2 rs3929*						
GG	160	114 (71.3)	1		1	
GC	95	67 (70.5)	0.91 (0.68–1.24)	0.554	0.94 (0.68–1.29)	0.696
CC	14	3 (21.4)	0.18 (0.06–0.57)	0.004	0.20 (0.06–0.63)	0.006
Trend test				0.009		0.023
CC	14	3 (21.4)	1		1	
GG+GC	255	181 (71.0)	5.31 (1.7–16.6)	0.004	4.98 (1.57−15.83)	0.006
Combined analysis^c^						
0 NUGs	12	3 (25.0)	1		1	
1 NUG	151	99 (65.6)	3.79 (1.20−11.96)	0.023	3.53 (1.10−11.36)	0.034
2 NUGs	106	82 (77.4)	5.42 (1.71−17.17)	0.004	5.46 (1.70−17.58)	0.004
Trend test				0.0003		0.0001

DFS, disease‐free survival; HCC, hepatocellular carcinoma; HR, hazards ratio; CI, confidence interval; NUG, number of unfavorable genotypes.

a Including relapse, metastasis and death.

b Adjusted by age, sex, ethnicity, HBsAg status, smoking status, drinking status, intact capsule, hepatic vein tumor thrombosis, cirrhosis and BCLC staging.

c Unfavorable genotypes were *SMAD3* rs11556090 AG+GG and *RBL2* rs3929 GG+GC.

### Combined effects and ROC curves for DFS prediction

To evaluate the joint effect of *SMAD3* rs11556090 and *RBL2* rs3929, we combined risk genotypes of rs11556090 CC and rs3929GG+GC into a single variable as the number of unfavorable genotypes (NUGs) (Table 5). The trend test indicated that increased NUG was associated with a shorter DFS regardless of univariate (trend test: *P *=* *0.0003) or multivariate analysis (trend test: *P *=* *0.0001). The Kaplan–Meier curves for depicting associations between NUG and DFS is also shown in Figure 1E. Using the ROC curves, we further evaluated predictive value of the unfavorable genotypes. The results demonstrated that, as classification of 1‐year DFS, the AUC was significantly increased from 68.4% to 74.1% (*P *=* *0.011), when adding NUG to the model including clinical variables (age, sex, ethnic, hepatitis B virus infection, smoking status, drinking status, capsule, portal vein tumor thrombosis, cirrhosis and BCLC staging) as classifiers (Fig. 1F).

### *In silico* functional validation

**Figure 2:**
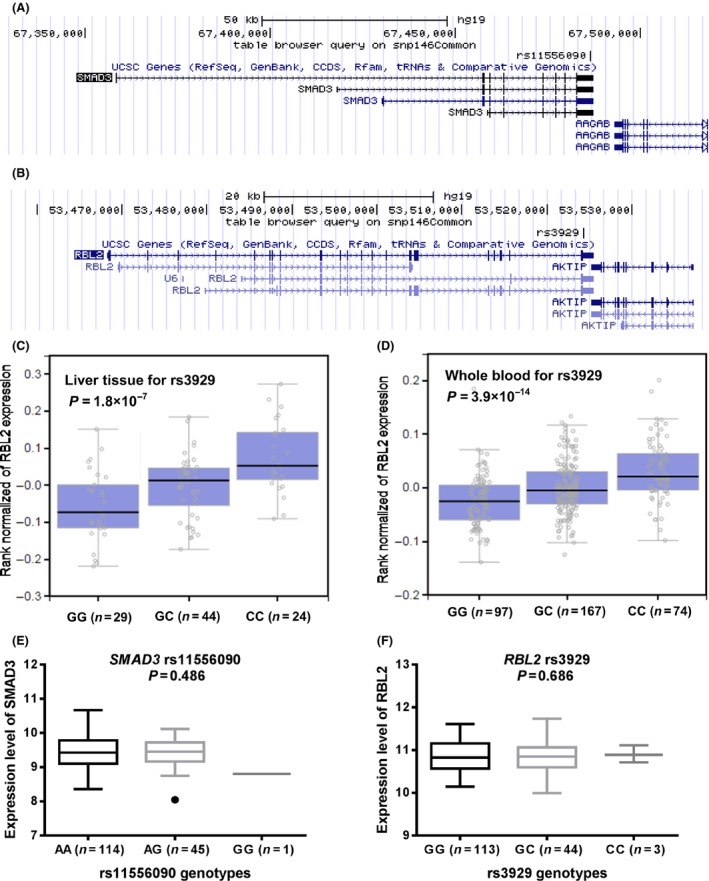
In *silico* functional validation of *SMAD3* rs11556090 and *RBL2* rs3929. Location‐map of rs11556090 (A) and rs3929 (B) in UCSC website (https://genome.ucsc.edu/); The expression quantitative trait loci analysis (eQTL) from the GTEx Portal (http://www.gtexportal.org/home/) for *RBL2* rs3929 in liver tissue (C) and the whole blood (D); eQTL analysis by using Asian population of HapMap3 data for rs11556090 (E) and rs3929 (F).

As mapped on the UCSC website (https://genome.ucsc.edu/), rs11556090 is located in exon 9, 3ʹUTR of *SMAD3*, while rs3929 is located in exon 22, 3ʹUTR of *RBL2* (Fig. 2A and B). Both of them were predicted to be miRNA binding sites of the corresponding genes by the SNPinfo. We further conducted the expression quantitative trait loci (eQTL) analysis by searching on the GTEx Portal (http://www.gtexportal.org/home/) and using data from the 1000 Genomes Project to examine the effects of SNPs on the mRNA expression levels of corresponding genes. We only found information about *RBL2* on GTEx Portal that the rs3929 C allele was significantly associated with increased expression levels of *RBL2* in liver tissues (*P *=* *1.8 × 10^−7^, Fig. 2C) and the whole blood (*P *=* *3.9 × 10^−14^, Fig. 2D) by general linear regression analysis. Data of Asian population (the Chinese and Japanese together) from the HapMap 3 Project showed no significant association for neither rs11556090 nor rs3929 with their corresponding gene expression levels (*P *=* *0.486 and 0.686, respectively, Fig. 2E and F).

## Discussion

In this hypothesis‐driven study, we identified *SMAD3* rs11556090 A>G and *RBL2* rs3929 G>C in the cell cycle pathway may independently or jointly modulate DFS of HCC patients. We also observed that combining with unfavorable genotypes of these loci significantly improved prediction performance of HCC survival in the model that included the known clinical prognostic variables. Furthermore, the results from the GTEx Portal demonstrated that the C allele was significantly associated with increasing expression levels of *RBL2*, suggesting that *RBL2* rs3929 may modulate DFS of HCC patients possibly through a mechanism of modulating gene expression.

Cell cycle control is an important process for DNA damage repair. When DNA damage occurs, cell cycle checkpoints are activated and cell cycle progression is paused, allowing cells sufficient time to repair the damage and determining whether cells will survive or die, which is controlled by the cell cycle pathway players 21. Previous studies have shown that the identified genes, *SMAD3* and *RBL2*, are important players in the cell cycle control. For example, *SMAD3* plays a critical part in the regulation of transforming growth factor‐β (TGF‐β), which is a potent inhibitor of cell cycle progression at the G1 phase 37. Likewise, *RBL2*, encoding one of the retinoblastoma family proteins (RBL2, also known as p130 and pRb2), is a key switch at the restriction point (R) and inhibits the S phase entry by physical combination with E2F transcription factor and actively represses gene transcription that regulates DNA synthesis in the S phase 38. Other studies reported that *SMAD3* functioned as a tumor suppressor in cancers of the stomach, 39, breast 40 and prostate 41, while *RBL2* functions as a tumor suppressor in cancers of the breasts, ovaries and endometrium 42. A similar function in HCC has also been illustrated for both of *SMAD3* and *RBL2* in the previous studies 43, 44, suggesting the significant function of *SMAD3* and *RBL2* as a tumor suppressor in HCC.

In this study, both of the two loci we identified were computationally predicted to be located in the microRNA‐binding sites by the SNPinfo. *SMAD3* rs11556090 is located in the binding site of hsa‐miR‐132, hsa‐miR‐188, hsa‐miR‐212, hsa‐miR‐30b, hsa‐miR‐337, hsa‐miR‐431, hsa‐miR‐532 and hsa‐miR‐9, while *RBL2* rs3929 was located only in has‐miR‐134. Surprisingly, the above‐mentioned miRNAs, except for has‐miR‐337 and has‐miR‐532, have been associated with development or survival of HCC 45, 46, 47, 48, 49, 50, 51. Therefore, it is likely that the underlying mechanisms of the two SNPs in modulating HCC survival are to regulate the transcription of proteins by affecting activities of miRNA‐binding. SNPs in the potential miRNA‐binding sites are suggested to be functional and always good candidates for causal variants of human disease occurrence or progress 52. Specifically, we found that the *RBL2* rs3929 C allele associated with a longer DFS was correlated with increased mRNA expression levels of *RBL2* in normal liver tissues and peripheral blood lymphocytes by using data from the GTEx Portal. These results are consistent with the characteristics of a tumor suppressor, although the majority of the donors for the GTEx project are of the white population. However, somatic mutations in *RBL2* were observed previously only in primary nasopharyngeal carcinomas, lung tumors, and Burkitt's lymphomas but not in HCC. Taken together, we can infer that *RBL2* rs3929 may be a functional and important variant for HCC progression.

The results also demonstrated a significant locus‐dosage effect in patients with a larger number of unfavorable genotypes, who had a shorter DFS, suggesting an interaction of *SMAD3* rs11556090 and *RBL2* rs3929 in the cell cycle pathway on HCC progress. The potential mechanism under the observed association is probably that both of SMAD3 and RBL2 are the key substrates of CDK4, which can control cell cycle progression from the G1 to S phase by phosphorylation of these proteins 37, 53. Importantly, The combination of the two loci significantly improved prediction performance of the model that included the known prognosis factors of HCC patients.

It should be noted that there were limitations of this study. The first limitation included restriction of the recruit population. We conducted the study only in the population from Guangxi Zhuang Autonomous Region, where the main cause of HCC is HBV infection 28. The results may not apply to other ethnic groups or disease etiologies, such as hepatitis C virus infection, nonalcoholic fatty liver disease and alcoholic liver disease. Secondly, the significance of *RBL2* rs3929 and *SMAD3* rs11556090 in the prognosis of HCC has been demonstrated in this study, however, it still lack epidemiology validation and biological mechanism confirmation, besides the significant eQTL results for *RBL3* rs3929 from the GTEx project. Finally, because of lack of more detailed clinical information, we did not efficiently evaluated the potential effects of different therapies after radical surgery resection on the outcomes of HCC patients, or their potential associations with the identified SNPs. Therefore, larger scale, more comprehensive and multi‐institutional epidemiology investigations and biological studies are warranted to further validate these results.

In conclusion, this study identified a prognostic role of *SMAD3* rs11556090 and *RBL2* rs3929 of the cell cycle pathway in HCC patients. Although both of *SMAD3* and *RBL2* are important in the control of cell cycle progression from the G1 to S phase, the interpretation of our findings should be cautious, until validated by mort patient cohorts and HCC cell lines.

## Conflict of Interest

The authors declare that they have no conflict of interest.
